# Music students’ workload, stress, and coping in higher education: Evidence-based policymaking

**DOI:** 10.3389/fpsyg.2022.846666

**Published:** 2022-07-15

**Authors:** Tuula Jääskeläinen

**Affiliations:** Sibelius Academy, University of the Arts Helsinki, Helsinki, Finland

**Keywords:** workload, stress, coping, music student, experience, intervention, policy recommendation, meta-narrative synthesis

## Abstract

Evidence-based policies are needed to support students as they cope with their experiences of workload and stress in higher music education. This subject was explored in the Music Student Workload Project as a collaboration between Finland and the United Kingdom in seven studies: (1) a theoretical study scrutinizing diverse higher music education systems in connection with equality and cultural reproduction; (2) a systematic review mapping international research on music students’ workload; (3) a methodological study discussing the transcendental phenomenological approach as a method for obtaining a meaningful understanding of music students’ experiences in higher education; (4) a qualitative study exploring music students’ workload experiences in connection with their meaningful engagement in music; (5) a mixed-method study shedding light on music students’ proactive coping styles in connection with workload and stress; (6) a mixed-method study examining music students’ experienced workload, stress, and livelihoods; and (7) a qualitative study exploring teachers’ ways of supporting music students’ workload and stress. The meta-narrative synthesis was conducted by triangulating the key elements of these studies to generate four actionable policy and intervention recommendations to inform educational policies and practices for supporting students in coping with workload and stress in higher music education: (1) support music students’ proactive coping skills; (2) find solutions to the unequal workload and stress experiences between low-income and well-off students, different genders, and different study programs; (3) ensure teachers’ continuing professional development, particularly in the learner-centered pedagogical approaches; and (4) invest resources for providing more longitudinal, cross-cultural, and interventional research investigating music students’ discipline-specific experiences of workload and stress.

## Introduction

Students’ workload in higher education affects their experiences in studying and wellbeing (Salmela-Aro and Read, 2017). Research in the context of higher education in Finland has shown alarming trends in the growth in students’ psychological distress, which may be connected to various and increased environmental and institutional demands on students (Oksanen et al., 2017). These pressures have become part of the current academic culture, in which neoliberalism has influenced many higher education policies (Gyamera and Burke, 2018), and which, in turn, has led to the situation where economic ideals guide students’ choices in studying, graduating, and working life (Johnston, 2011). Although egalitarianism as an element of social progression has become a common and self-evident concept in educational policies (Kalalahti and Varjo, 2012), current educational systems with business-minded administrations and expectations for productivity and efficiency may nevertheless include elements that enhance or even introduce new inequalities (Reay, 2017). In the United Kingdom, the implementation of a neoliberalization policy agenda brought with it increased tuition fees for public higher education (Maisuria, 2014). Higher education in Finland did not have a tuition fee system before 2010 when the new university law separated higher education institutions from the state and gave them “an independent legal personality, either as public corporations or as foundations under private law” (Pekkola, 2009, p. 2). This neoliberal university reform seems to have strengthened the reproduction of social inequalities between genders and between classes and the polarization between privileged and less privileged individuals within the institution, as it involves the culturally and situationally specific forms of ideal academic boasting (Lund, 2020). For example, within this neoliberal reform, “students and workers are encouraged to internalise the values and presuppositions of the culture of innovation, to consider their work projects as their personal projects, to identify their advancement at the professional markets with their personal growth, to think, feel, perceive and sense in terms dictated by the economy” (Pekkola, 2009, p. 4). When looking particularly at higher music education policies and practices in Western countries, Bull (2020) argues that the historical roots in cultural reproduction have strengthened the middle class, maintained gendered patterns, and legitimized hierarchical, competitive, and exclusive practices in music education. Therefore, to pursue broader progress in educational equality, it is important to make public the evidence for inequalities in educational policies and practices. One way to produce this kind of evidence is to investigate music students’ experiences of their workload and stress as they try to cope with the demands of higher education studies.

Student workload is a topic that has already been researched widely in higher education contexts (e.g., Chambers, 1992; Lockwood, 1999; Marsh, 2001; Kember, 2004; Bowyer, 2012). Previous research also shows how it is possible to support students in their coping with study workloads, for example with teachers’ professional development work in their universities (Giles, 2009); assessment that supports learning processes (Hernesniemi et al., 2017); and constructive cooperative teaching (Kember and Leung, 2006). Reducing students’ perceived overload is important because an overly heavy workload can have a negative effect on students’ wellbeing and success in studying (Hernesniemi et al., 2017). On the other hand, teachers can increase both the demands and quality of learning without increasing students’ perceived workload if teachers are able to maintain a cooperative atmosphere in the class (Kember and Leung, 2006). Scholars investigating students’ workload emphasize that more research is needed, in particular about students’ time and stress management and how increasing coping skills can affect students’ ability to deal with workload, learning, distress, and burnout in higher education (Jacobs and Dodd, 2003; Kember, 2004; Amirkhan and Kofman, 2018).

Although exploring students’ perceptions is a common approach to investigating their workload (Kember, 2004; Kember and Leung, 2006), the research on students’ experiences of their workload (*experienced workload*), in particular in the field of music, can provide deeper knowledge for developing policies when higher music education institutions are seeking ways to support music students as well as possible. Indeed, in the higher education context, music students experience specific field-related challenges when compared to that of students in other disciplines. Performance anxiety, perfectionism, and career concerns are common sources of stress in studying music (e.g., Bernhard, 2007). Music students also suffer from painful musculoskeletal conditions and other health issues in studying music (e.g., Ginsborg et al., 2009), and there have been reported differences in experienced stress, particularly between genders, and in mood, bodily tensions, and somatic symptoms between music students studying in different programs (Zetterberg et al., 1998). Sudden and unexpected changes in learning circumstances, such as that which the COVID-19 pandemic has caused, may also affect music students’ wellbeing (Habe et al., 2021), practice habits, and behaviors, as well as their everyday lives (Rosset et al., 2021).

In the context of studying music, workload is often connected to the negative consequences that difficult or unmanageable study situations may result in for music students, such as burnout (Bernhard, 2007) and mental illness (Koops and Kuebel, 2021). When aiming to support music students’ ability to cope with workload, it is interesting to take note of their experiences of meaningful engagement in music, such as their passion and love for music, because they can be positive sources of workload for them (Park et al., 2007). It is also important to explore which environmental and intra-individual factors may be connected to music students’ experienced workload in higher education. Thus, expanding our knowledge of music students’ experiences in studying music is essential to understanding both the stressors and the resources associated with their workload, and to supporting the students’ health, wellbeing, and learning (Koops and Kuebel, 2021). Moreover, Bresler (2005) argues that research focusing on lived experiences of musicianship and studying music have the potential to contribute to research methodologies in general, not only in music education but also in the social sciences and educational research. Research-based evidence on music students’ experiences, when connected to the development of pedagogical practices in higher music education, can support the efforts of teachers and administrations to gain a deeper understanding of the diversity in student populations and the individual circumstances of their students (see, e.g., Harrison, 2014). This kind of approach may help educational institutions to improve their support systems for students, to develop teaching and learning environments, and to advance educational policies.

Because research-based findings regarding students’ experienced workload in higher music education are still lacking to a great extent, the Music Student Workload (MSW) Project was established in 2017 as a research collaboration between University of the Arts Helsinki in Finland and Royal Northern College of Music in the United Kingdom. The primary aim was to investigate music students’ experiences of workload, stress, and coping in higher education. The researchers in this project were interested in all aspects of music students’ workload during their years of study in higher education, such as the nature, meaning, and components of their workload. The interest was also in more concrete aspects of student workload while studying (such as attendance at lectures, rehearsals, and practice sessions), and also at other times (such as paid and unpaid work). Both negative aspects, such as burnout, and positive aspects, such as flow, were considered to be workload components. In particular, the interest was in the quality of music students’ workload and its overall consequences. Therefore, the aim was to investigate how the students themselves experience their workload rather than measuring evidence for its quantity (such as time spent studying, completed credits, and grades). The data were collected in seven higher music education institutions in Finland and the United Kingdom. Over 4 years, a total of seven studies were conducted in the MSW Project (see study characteristics in Table 1): (1) a theoretical study scrutinizing diverse higher music education systems in connection with equality and cultural reproduction (Jääskeläinen, 2021); (2) a systematic review mapping international research on music students’ workload (Jääskeläinen et al., 2022b); (3) a methodological study discussing the transcendental phenomenological approach as a method for obtaining a meaningful understanding of music students’ experiences in higher education (Jääskeläinen, 2022b); (4) a qualitative study exploring music students’ workload experiences in connection with their meaningful engagement in music (Jääskeläinen, 2022a); (5) a mixed-method study shedding light on music students’ proactive coping styles in connection with workload and stress (Jääskeläinen et al., 2022a); (6) a mixed-method study examining music students’ experienced workload, stress, and livelihoods (Jääskeläinen et al., 2020); and (7) a qualitative study exploring teachers’ ways of supporting music students’ workload and stress (Jääskeläinen and López-Íñiguez, 2022).

**Table 1:** Study characteristics.

Study	Study design	Main research question(s)	Data collection	Sample	Analysis	Main findings and/or results
1. Tuition fees, entrance examinations and misconceptions about equity in higher music education (Jääskeläinen, 2021)	Theoretical study	How are equality and cultural reproduction connected to tuition fees and entrance examinations in higher music education institutions?	Not applicable	Not applicable	Not applicable	Tuition fee systems in higher music education institutions may aim at improving equality, equity, and justice for students but they can also enhance inequalities. Traditions in the field of music have created a strong culture of entrance examinations that is not easy to change because the roots of cultural reproduction are deeply embedded in educational systems.
2. Music students’ experienced workload in higher education: A systematic review and recommendations for good practice (Jääskeläinen et al., 2022b)	Systematic literature review	1. What factors have an impact on students’ experienced workload?	Systematic literature search	12 studies on students’ experiences of workload in higher education and 17 studies on music students’ experiences of workload in studying music	Extended meta-ethnography	A total of 24 recommendations for good practice to
						(a) increase music students’ ability to cope with their workload,
		2. What are music students’ experiences of workload in relation to their studies?				(b) provide tools for teachers to support music students to manage and cope with workload, and
						(c) develop learner-centered environments in higher music education
3. Using a transcendental phenomenological approach as a model to obtain a meaningful understanding of music students’ experienced workload in higher education (Jääskeläinen, 2022b)	Methodological study	In what ways can a transcendental phenomenological research approach offer insights into music students’ lived experiences in higher education?	Not applicable	Not applicable	Not applicable	Transcendental phenomenological research approach provides a practical model for addressing music students’ unique and meaningful experiences in relation to future administrative and teaching developments in higher music education institutions, such as processing and incorporating students’ feedback into improvements in teaching and learning environments.
4. “Music is my life”: Examining the connections between music students’ workload experiences in higher education and meaningful engagement in music (Jääskeläinen, 2022a)	Qualitative study	What does engagement in music mean to 29 music students in higher education in Finland and the United Kingdom, when they reflect on the experiences of their studies and workload?	Interviews	29 music students in higher education in Finland and the United Kingdom	Transcendental phenomenology approach	Various holistic workload experiences:
						(1) the music students’ intense and complex experiences
						(2) their development as musicians
						(3) their creative self-expression
						(4) their interactions with others
						(5) their personal growth and coping approaches
						(6) their transcendental musical experiences
5. Experienced workload, stress, and coping among professional students in higher music education: An explanatory mixed-method study in Finland and the United Kingdom (Jääskeläinen et al., 2022a)	Multi-strategy study	How do professional students in higher music education in Finland and the United Kingdom experience workload and stress and use proactive coping styles?	Questionnaire and interviews	155 music students in higher education in Finland and the United Kingdom	Frequentist statistics and transcendental phenomenology approach	Statistically significant differences among music students in genre groups and study programs in relation to experienced study workload; in genders, genre groups, and study programs in relation to experienced stress; and in genders in relation to use of coping styles. Study workload is a significant predictor of stress. Music students have their issues and ways to cope with workload and stress.
6. Music students’ experienced workload, livelihoods and stress in higher education in Finland and the United Kingdom (Jääskeläinen et al., 2020)	Multi-strategy study	What are the predictors and determinants associated with music students’ experiences of workload in relation to their livelihoods and stress in higher education?	Questionnaire and interviews	155 music students in higher education in Finland and the United Kingdom	Bayesian statistics and transcendental phenomenology approach	There is a need to pay attention to workload especially with undergraduate-level students, to stress especially regarding junior and doctoral-level study, and to particular areas of study, especially music education. Working while studying has an impact on music students’ workload and stress, thus affecting students’ relationship with studying and being a musician.
7. Tools for teachers to support music students in managing and coping with their workload in higher education (Jääskeläinen and López-Íñiguez, 2022)	Qualitative study	What constructive tools for teachers can support music students in managing and coping with their experienced workload in higher education?	Questionnaire and interviews	155 music students in higher education in Finland and the United Kingdom	Transcendental phenomenology approach	A total of 43 constructive tools for teachers based on the music students’ experiences.in the interactions with teachers concerning
						(1) the structure of workload
						(2) a music student’s individual workload
						(3) workload relating to teaching and learning environments
						(4) psychological and physiological issues.

**Figure 1:**
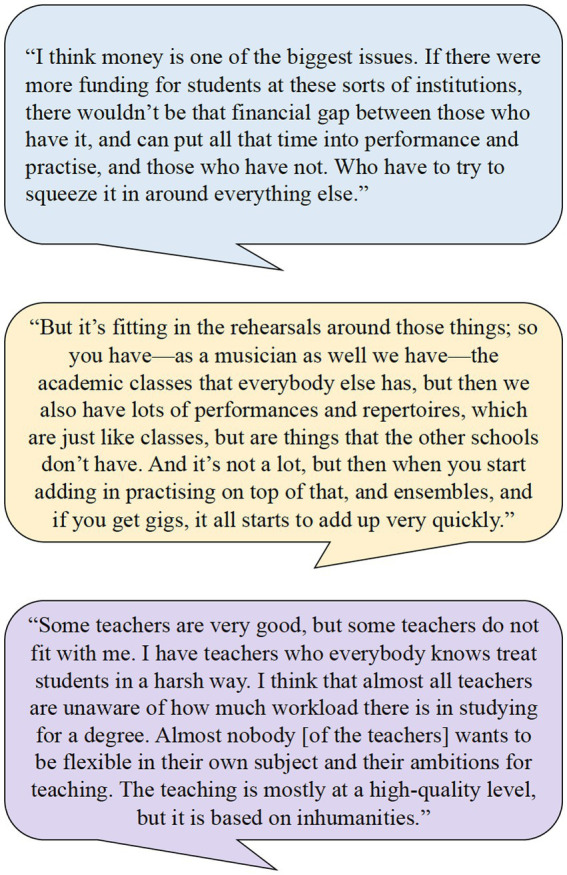
Research participants in the MSW Project telling about music students’ experienced workload.

In order to illustrate how experienced workload was approached in the MSW Project when listening to music students’ lived experiences, Figure 1 shows some quotations from research participants. The first experience is about a music student’s coping with their workload, the second experience is about workload relating to the teaching and learning environments in the field of music, and the third experience is about a music student’s workload in their interactions with teachers.

The objective of the present study is to bring together new and previous research on music students’ workload in higher education by synthesizing the findings and results of the MSW Project through a meta-narrative approach for generating evidence-based policy and intervention recommendations. More specifically, the aims of the study are to:

1. map previous and new research on music students’ experienced workload,
2. review the key elements of the findings and results from the seven studies in the MSW Project to provide policy and intervention recommendations, and
3. triangulate these key elements to build a synthesis of policy and intervention recommendations for supporting music students in coping with workload and stress in higher music education.

## Materials and methods

### Meta-narrative approach

The MSW Project incorporated diverse approaches to generating evidence about music students’ experienced workload. In order to synthesize the heterogeneous evidence that had been studied in various ways in the seven studies, the MSW Project team chose to use an adaptation of the meta-narrative approach (originally developed by Greenhalgh et al., 2004, 2005). A meta-narrative synthesis develops an overarching narrative through story lines that show how research for a particular topic evolves over time and throughout different disciplines, leading to key insights (Greenhalgh et al., 2005). The six guiding principles of the meta-narrative method were followed (Greenhalgh et al., 2005; Wong et al., 2013): (1) pragmatism: the included information was reviewed according to its usefulness to the policy and intervention recommendations; (2) pluralism: the topic was considered from multiple perspectives in the seven studies; (3) historicity: the included information was presented according to its development over time, as there were no date restrictions on the systematic literature review; (4) contestation: conflicting information was used to generate higher-order insights; for example, data were collected in two countries with different higher education systems, and both frequentist and Bayesian statistics were used in the statistical analyses; (5) reflexivity: there was continual reflection on the studies in the MSW Project team, and the findings and results were also presented and discussed in several international conferences; and (6) peer-review: each of the seven studies went through an international, blinded peer-review process before publication in research journals.

We adapted the phases of the meta-narrative review according to Greenhalgh et al. (2004, 2005), including (1) a planning phase, where a MSW Project team—comprising the author of the present study as a principal investigator and three co-researchers/supervisors in Finland and one co-researcher/supervisor in the United Kingdom—was compiled; (2) a search phase, where previous research was searched with a systematic literature review and complemented with new research by conducting one theoretical, one methodological, and four empirical studies; (3) a mapping phase, where the findings and results from these seven studies were used to establish the key elements of music students’ experienced workload, stress, and coping in higher education; (4) an appraisal phase, where the key elements were critically appraised for their validity and relevance to policy and intervention recommendations; (5) a synthesis phase, where a narrative account of included key elements for policy and intervention recommendations was prepared through a triangulation; and (6) a recommendations phase, where the findings from the triangulated key elements were summarized and policy and intervention recommendations were made for supporting music students in coping with workload and stress in higher music education.

## Results

### A planning phase

The planning phase started in 2017 when the research collaboration between University of the Arts Helsinki in Finland and Royal Northern College of Music in the United Kingdom was established and the MSW Project team was compiled. The research plan was written by the first author and critically commented by the three co-researchers/supervisors in Finland and one co-researcher/supervisor in the United Kingdom. The ethical evaluations were carried out for the research by the University of the Arts Helsinki Ethics Committee in 2018 and the Conservatoires United Kingdom Research Ethics Committee in 2019, and the statements were favorable. The research permissions were obtained from seven randomly chosen participating institutions in Finland and the United Kingdom.

### A search phase

**Figure 2:**
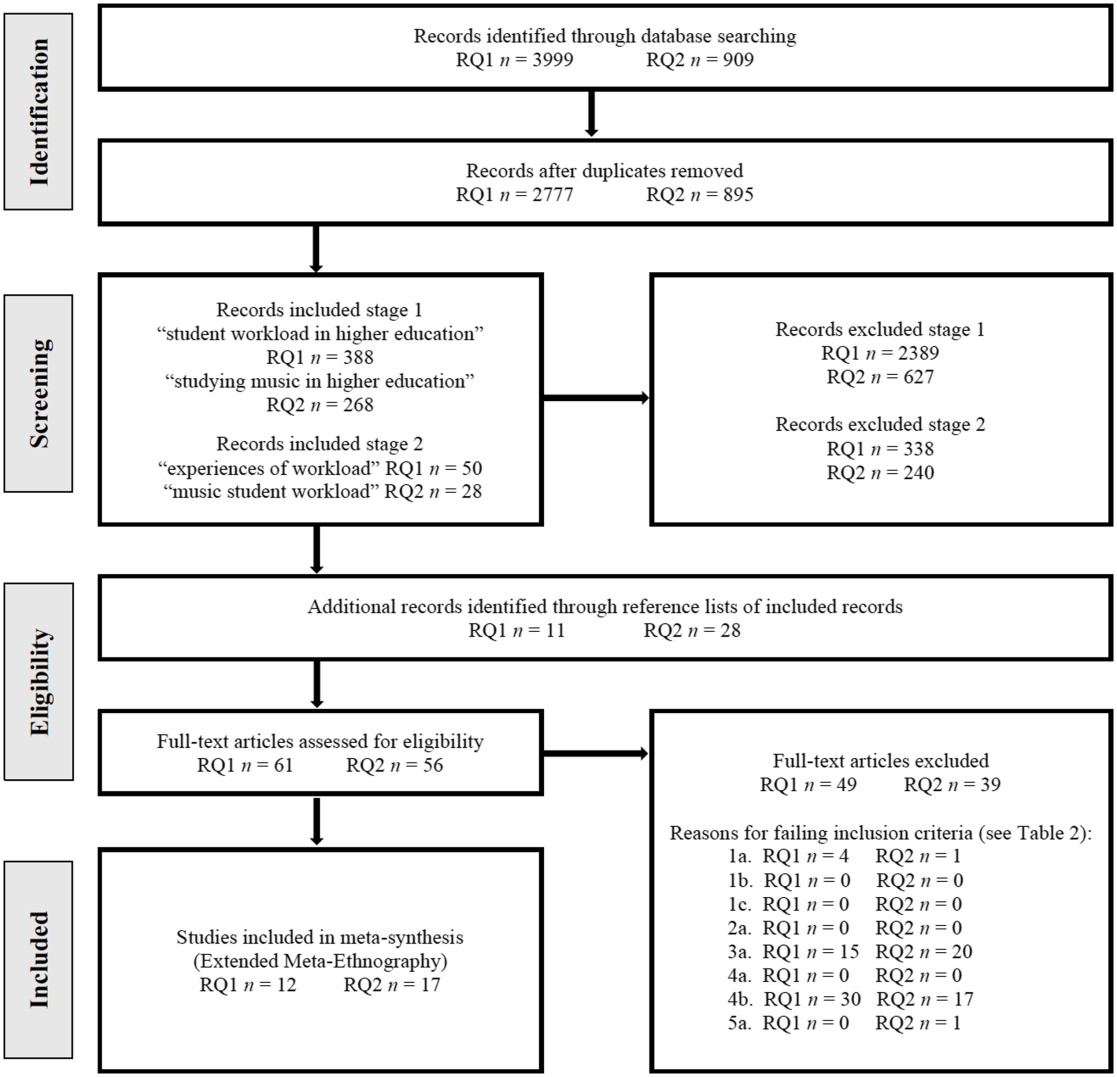
Process of selecting studies grouped by two research questions (RQ) in PRISMA flowchart 2009 (Moher et al., 2009).

When starting the search phase, it was important to take a closer look at the similarities and differences of higher music education systems in different countries—particularly because the MSW Project was a cross-cultural research project—and to understand how these systems might be connected to educational equality, equity, and justice, and cultural reproduction. These topics were discussed in the theoretical study (i.e., Jääskeläinen, 2021), which was conducted in 2018. In the same year, we prepared the protocol for the systematic review (registered in PROSPERO CRD42020140497) and performed the systematic search of literature in 23 electronic databases and 19 music research journals. After screening the references which were found in the searches, 29 qualitative, quantitative, and multi-strategy studies fulfilled the inclusion criteria. These studies were conducted in the United States (*n* = 8), the United Kingdom (*n* = 5), Australia (*n* = 3), Finland (*n* = 3), Hong Kong (*n* = 2), New Zealand (*n* = 2), Belgium (*n* = 1), China (*n* = 1), Italy (*n* = 1), Pakistan (*n* = 1), Puerto Rico (*n* = 1), Spain (*n* = 1), and Sweden (*n* = 1). A total of 13,596 students took part in the 29 studies, of whom 2,261 were music students. Figure 2 shows the process of selecting studies following the guidelines of Preferred Reporting Items for Systematic Reviews (PRISMA). After selecting the studies, we conducted an extended meta-ethnography (EME)—a method of systematic review developed by Booker (2010)—to create a synthesis. The process and results were reported in the systematic review (i.e., Jääskeläinen et al., 2022b).

The theoretical study and systematic review were complemented with new methodological and empirical research. For the empirical studies, the assessment instrument—the Workload, Stress, and Coping questionnaire—was created by combining and adapting sections from two validated questionnaires from the learning sciences. The first instrument was the standardized study workload and stress section of the Learn questionnaire used in the Finnish higher education context (i.e., Parpala and Lindblom-Ylänne, 2012). The second instrument was the Proactive Coping Inventory for Adolescents (PCI-A), developed in Canadian higher education (i.e., Greenglass et al., 2008). The Workload, Stress, and Coping questionnaire also included demographic items and open-ended questions about workload, stress, coping, and the students’ interactive experiences with teachers. The experienced study workload scale included five items. The scale assessed students’ workload experiences when considering their studies as a whole, in their main subject (or principal) studies (e.g., “I must work very hard with my main subject studies”). A single item assessed students’ current feelings of experienced stress. The proactive coping section included seven different scales assessing proactive coping styles, with a total of 55 items (e.g., “I plan my strategies to change the situation before I act”): proactive coping, reflective coping, strategic planning, preventive coping, instrumental support seeking, emotional support seeking, and avoidance coping (PCI-A, see Greenglass et al., 2008).

The data were gathered online through an institutional Surveypal-questionnaire during the spring term of 2019. We randomly selected seven university-level music institutions in Finland and the United Kingdom. We sent the invitation to participate in the research via student email lists, thus potentially reaching over 7,000 music students. A total of 155 music students from five different institutions (including a total of 5,900 music students) completed the questionnaire in Finland (*n* = 108) and the United Kingdom (*n* = 47). The total response rate was relatively low (9% in Finland and 1% in the United Kingdom), which is quite common for online surveys among students, because of the survey fatigue they are typically exposed to (Porter et al., 2004). Of the questionnaire respondents, 29 music students volunteered to participate in the interviews. Semi-structured in-depth interviews were conducted, either in contact meetings or remotely, ranging from 30 to 90 min. The topics consisted of questions that encouraged students to reflect on their workload, stress, and coping as professional students in higher music education. Demographic characteristics of all participants are given in Table 2.

**Table 2:** Demographic characteristics of all participants in the sample (*N* = 155).

Background	%	Main subject studies	%
Country		Genre group	
Finland	69.7	Classical music (UG or PG)	43.2
United Kingdom	30.3	Music education (UG or PG)	24.5
Gender		Other genres	32.3
Female	68.0	Study program	
Male	30.1	Classical string	13.5
Non-binary gender	2.0	Classical wind	9.7
University level		Classical piano	6.5
Undergraduate (UG)	52.9	Classical early music	3.2
Postgraduate (PG)	42.6	Classical other instruments	3.2
Other (junior or doctoral)	4.5	Classical voice and opera	7.1
		Music education	24.5
Interview participants (*n* = 29)	18.7	Composition	7.7
Finland (*n* = 20)		Church music	12.3
United Kingdom (*n* = 9)		Folk and global music	4.5
Female (*n* = 21)		Other programs	3.9
Male (*n* = 8)		Doctoral programs	3.9

**Figure 3:**
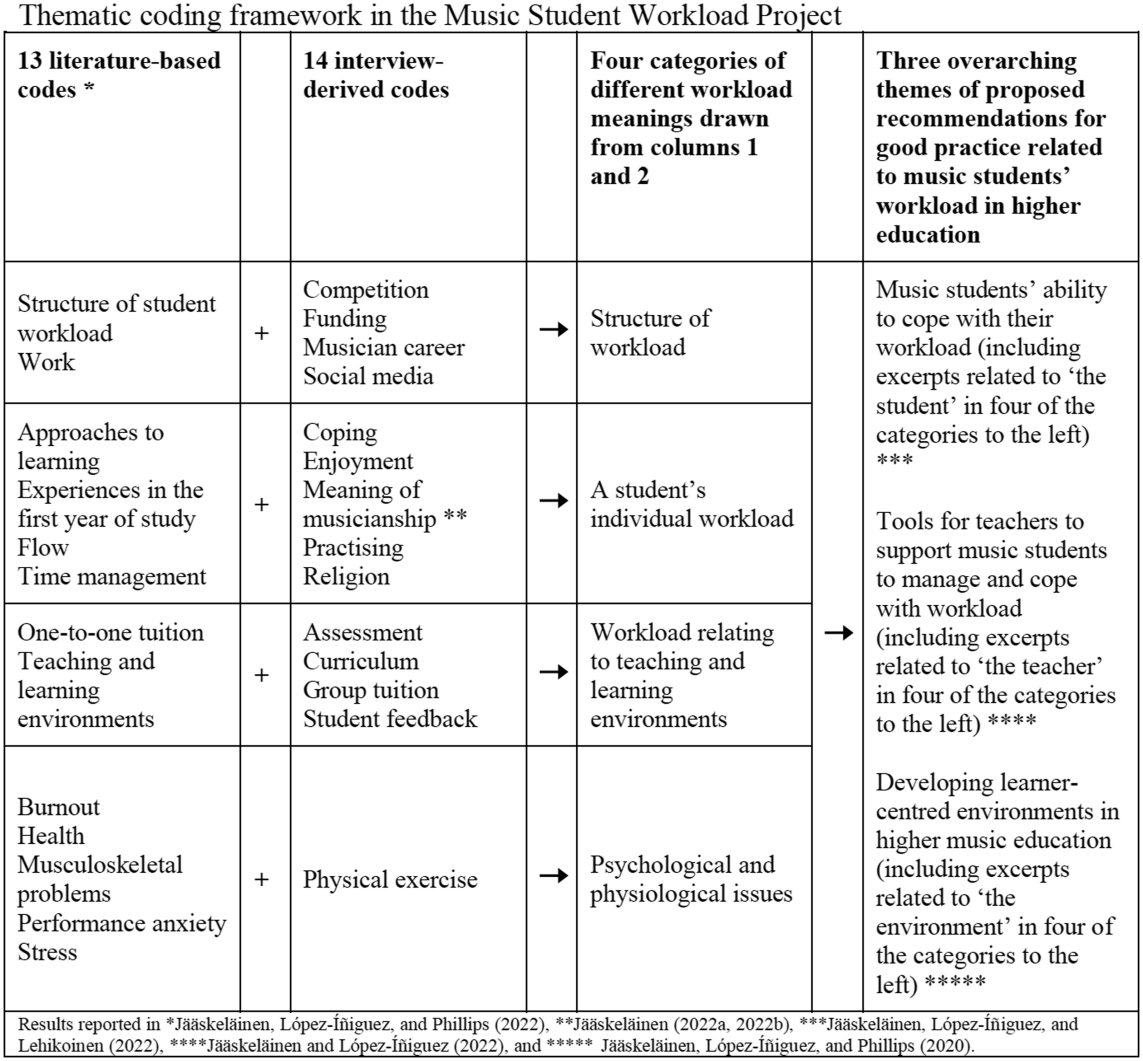
Music Student Workload Project’s thematic coding framework.

The quantitative data in the questionnaire were analyzed with SPSS using frequentist statistics and with RStudio by using Bayesian statistics. Atlas.ti was used to code and analyze the qualitative data concerning the answers to the open-ended questions in the questionnaire and transcribed interviews, by adapting the analytical process of transcendental phenomenology (Moustakas, 1994). The analysis was performed by the author in collaboration with one co-researcher/supervisor who specializes in the psychology of music education, who coded 5% of the qualitative data in order to enhance the reliability and trustworthiness of the research process. The inter-rater agreement of the two independent coding choices was calculated by using Holsti’s (1969) method and Krippendorff’s (1980) alpha, and was favorably calculated as 0.924 and 0.918, respectively, with both values indicating very high levels of reliability. A thematic coding framework (see Figure 3) was built based on the 13 codes, four categories, and three overarching themes that were derived from the deductive systematic review (Jääskeläinen et al., 2022b). Next, an additional 14 themes that were extracted from the interview data using inductive analysis were added to the framework, to contextualize the music students’ responses relative to the existing literature on workload experiences in higher music education. The analysis continued through the process of horizonalization, in which all the students’ responses that are relevant to their workload were listed, grouped, and coded for each individual participant’s data. The procedure of using transcendental phenomenological approach was presented and discussed more in detail in the methodological study (i.e., Jääskeläinen, 2022b). At this point, the excerpts which were coded as *meaning of musicianship*, were explored more deeply, because *engaging in music* seemed to be a topic for which the interview participants expressed lots of positive emotions while speaking about their workload experiences. These findings were reported in the empirical study (i.e., Jääskeläinen, 2022a).

Next, all the coded expressions were grouped into four categories and three overarching themes based on the context of the music student’s experienced workload, and for the empirical studies, we continued the analysis with the extracts linked to each of the overarching themes: (1) *student* in Jääskeläinen et al. (2022a), (2) *environment* in Jääskeläinen et al. (2020), and (3) *teacher* in Jääskeläinen and López-Íñiguez (2022). The Finnish participants’ quotes were translated from Finnish into English by the author, who speaks both languages, and refined in English by a native language editor.

### A mapping phase

In the mapping phase, the findings and results from these seven studies were used to establish the key elements of music students’ experienced workload, stress, and coping in higher education. Next, the key elements are presented in each MSW Project’s study. The findings of the theoretical study (i.e., Jääskeläinen, 2021) focused on the comparison of higher music education systems in three different countries. A critical debate about the role and impact of tuition fees and entrance examination systems is needed in higher music education, and this discussion should be situated within an evaluation of contemporary educational policy trends. According to Bull (2020), crucial topics include: (1) social inequalities; (2) genres, class, genders, and race, (3) sexual, emotional, and physical abuse in the field of music; and (4) creative collaboration with other social groups. Moreover, it is vital to listen to music students talk about their joys and concerns, such as coping with workload and stress, and to integrate their voices into the music institutions’ developmental work. Addressing and sharing individual experiences of inequality and oppression in educational systems can make more inclusive education possible (Reay, 2017). Reimer (2007) argues that although inequities and injustices are not going to disappear in music education, it is crucial to pursue broader equality, equity, and justice. All efforts, even those with modest positive results or resulting in only slight progress, are valuable.

The systematic review (i.e., Jääskeläinen et al., 2022b) revealed three overarching themes, offering a total of 24 recommendations for good practice for students, teachers, administrators, and student health and wellbeing services, as to how to deal with music students’ workload. As a result, a framework of music students’ experienced workload was constructed based on three contexts where developmental actions could be recommended in higher music education: (1) music students’ studying and coping strategies, (2) teachers’ interaction with music students; and (3) aspects in teaching and learning environments, such as university institution and livelihoods.

The transcendental phenomenological approach was an essential method for the MSW Project. That is why a closer look was taken at it in the methodological study (i.e., Jääskeläinen, 2022b). This study provided a detailed description of how music students’ lived experiences can be approached and analyzed through transcendental phenomenology, in order to obtain a meaningful understanding of music students’ experienced workload.

**Figure 4:**
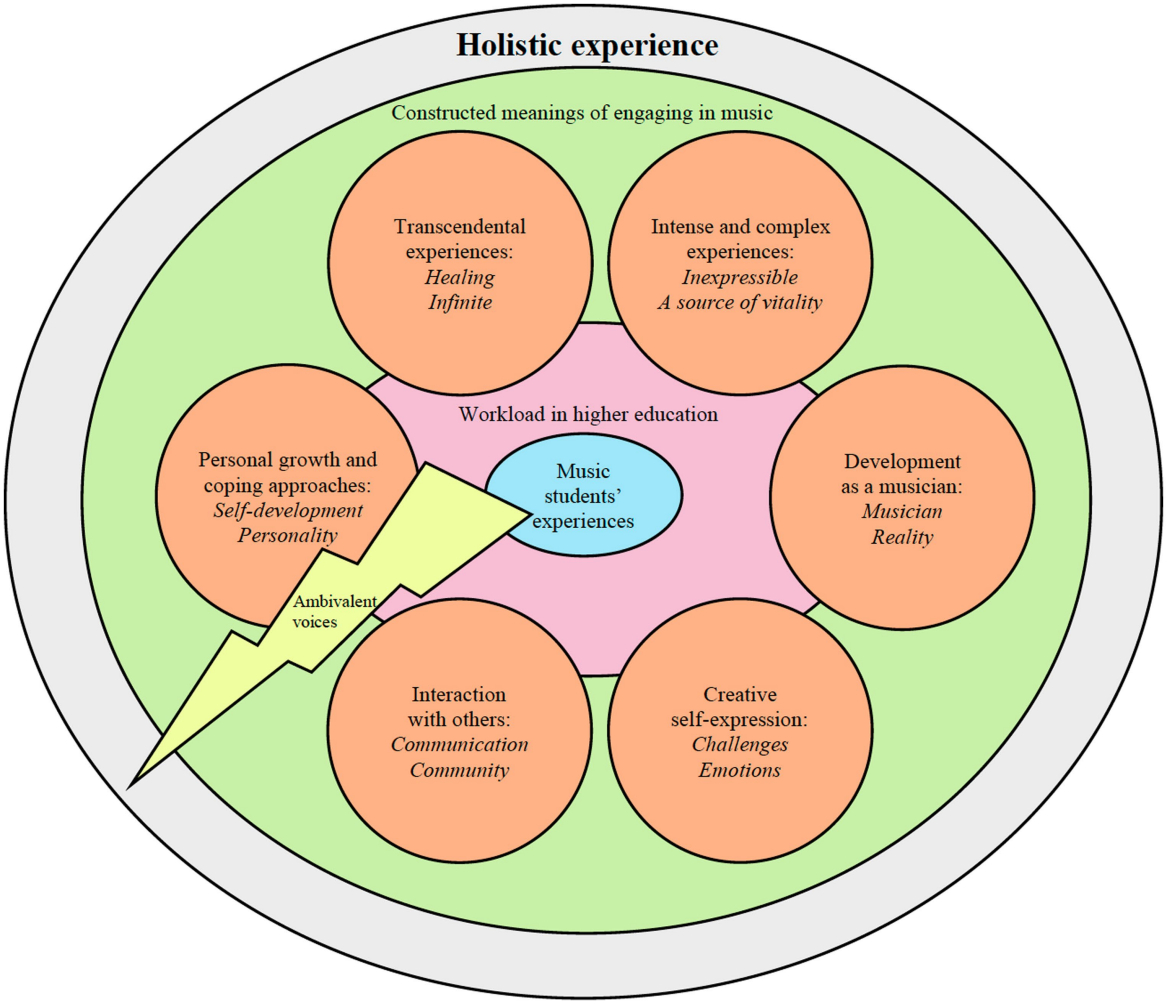
Music students’ workload experiences in higher education connected to their constructed meanings of engaging in music.

In the first empirical study (i.e., Jääskeläinen, 2022a), music students’ experiences of workload, which were coded as *meaning of musicianship*, were explored more deeply. When researching students’ workload, the examined themes often emphasize negative aspects of workload, such as overload, stress, burnout, and mental illness. However, workload also includes positive aspects that are as important as the negative aspects, such as music students’ meaningful *engagement in music*, that can help students find a way to continue their studies. In Figure 4, the findings of this study show how music students’ workload experiences in higher education are connected to their constructed meanings of engaging in music. These connections as a whole can be understood as various holistic experiences that comprise the following constructed meanings of musical engagement: (1) the students’ intense and complex experiences, (2) their development as musicians, (3) their creative self-expression, (4) their interactions with others, (5) their personal growth and coping approaches, and (6) their transcendental musical experiences. In addition, the several students who expressed their ambivalent views (illustrated by the lightning in Figure 4) have been included next to their constructions of Personal growth and coping approaches, in order to highlight how listening to the students’ different views can provide important knowledge on how to better nurture these students’ engagement in music throughout their studies in higher music education. The students’ emotional reactions as they spoke about the meaning of engaging in music have shown that music is a source of vitality to them, and, furthermore, that one’s experiences of engaging in music is a complex phenomenon that many of the participants found difficult to describe. This participant’s quotation illustrates an example of a music student’s experience in the Personal growth and coping approaches:

*“Yeah, music to me is everything. Yeah, I guess it [music] is my life. I would not be who I am today without music*.*”*

In the second empirical study (i.e., Jääskeläinen et al., 2022a), Figure 5 shows the integration of the qualitative findings with the quantitative results. The results indicate that there are statistically significant differences among music students in different genre groups and study programs in relation to experienced study workload; in genders, genre groups, and study programs in relation to experienced stress; and in genders in relation to the use of proactive coping style. Music students’ study workload is a significant predictor of stress. However, proactive coping and strategic planning can be used to reduce this stress, because they predict stress negatively. Professional music students have their own issues and ways to cope with workload and stress concerning, for example, working alongside studying, competing with peer-students, handling information on social media, and finding support for music-specific physical and psychological problems. This participant’s quotation illustrates a music student’s experience in using an emotional support-seeking coping style:

**Figure 5:**
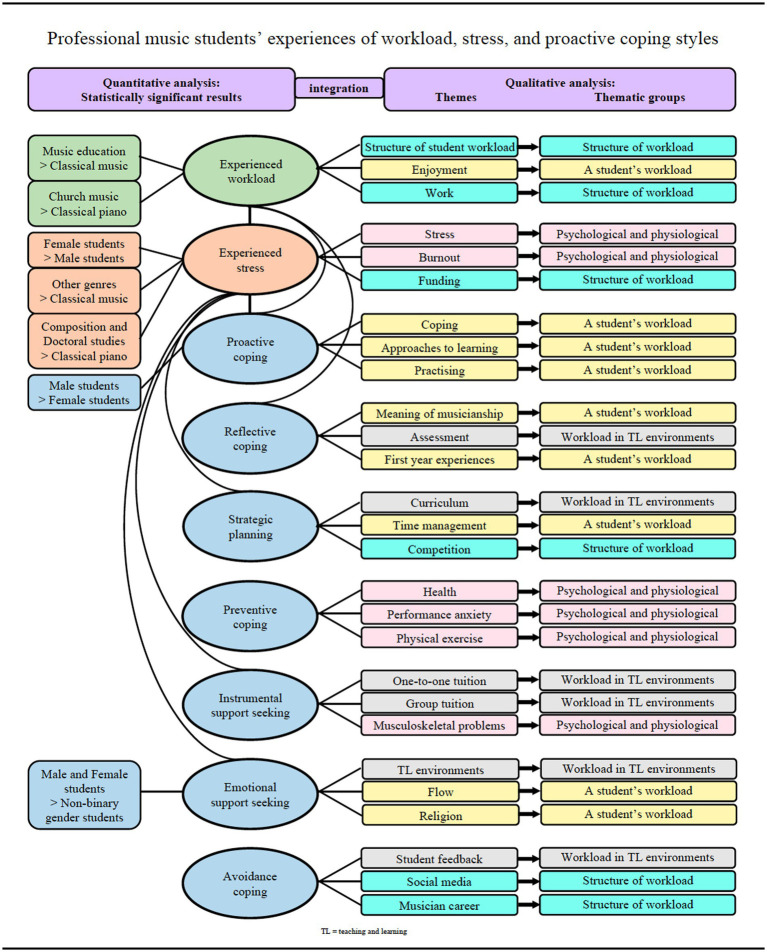
Integration of the results of the quantitative analysis and the findings of the qualitative analysis concerning professional music students’ experiences of workload, stress, and proactive coping styles.

*“I got peer support from the other students. You hear that very many of your peer students have also had enormous stress. It has been very encouraging to notice that people also talk openly to each other about these issues. And also, for example, recommend to each other that there is this kind of study counsellor where you can go*.*”*

In the third empirical study (i.e., Jääskeläinen et al., 2020), Figure 6 shows the full model of music students’ experienced main subject workload, which indicates that, when connected to these characteristics of livelihoods, stress was the strongest predictor of workload. There was also an effect for funding, such that music students with partial funding or no funding at all were less likely to report experiencing workload than students with full funding. Work related to music had a greater effect than work not related to music, but the total amount of work undertaken alongside a student’s studies had a negligible effect on workload. Female music students were likely to report experiencing more workload than male or non-binary gender students. The level of the university studies, in general, had a relatively small impact on the results; however, undergraduate music students were likely to report experiencing more workload than postgraduate students or junior and doctoral students. The music genre studied had little influence on the level of experienced workload, although studying music education had a greater effect compared to other genres, whereas the classical music genre had a negligible effect. Figure 7 shows the full model of music students’ experienced stress, which indicates that, when connected to the characteristics of livelihoods, music students in the United Kingdom are more likely to report experiencing stress than music students in Finland. Workload was the strongest predictor of stress in the full model. There was also a noticeable effect for gender, such that female music students were more likely to report experiencing stress than male students. With the non-binary gender, there was a positive effect on stress, which was contradictory to the negligible effect on workload in the previous full model. Both work not related to music and the total amount of work undertaken alongside studying had a small effect, but work related to music did not have an influence on stress. Junior or doctoral music students were much more likely to report stress than postgraduate students or undergraduate students, which contradicts the full workload model, in which being an undergraduate student had more influence on workload. The use of loans had no effect on stress (nor on workload in the previous model). This participant’s quotation illustrates a music student’s workload experience relating to teaching and learning environments:

**Figure 6:**
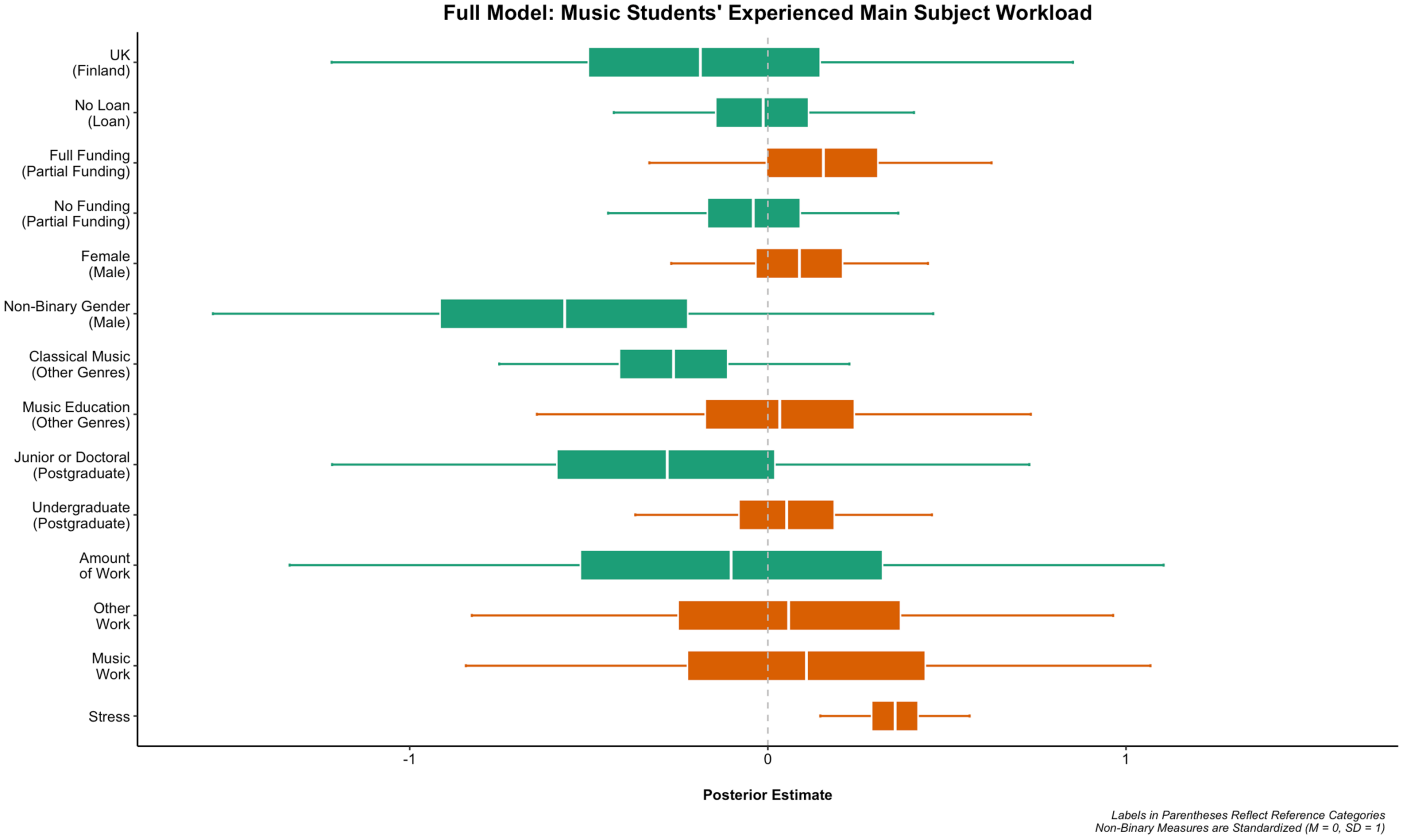
Population-level predictors of experienced main subject workload, derived from a Bayesian mixed-effect probit regression. The boxes indicate 50% posterior intervals, and the lines indicate 95% posterior intervals. With binary items, the left-hand side boxes indicate a smaller effect on workload than in the reference group in brackets, and the right-hand side boxes indicate a greater effect on workload than in the reference group in brackets. With the working and stress items (the four bottom items on the figure), the left-hand side boxes indicate a negligible effect on workload and the right-hand side boxes indicate a greater effect on workload the nearer the box is to the right-hand side.

**Figure 7:**
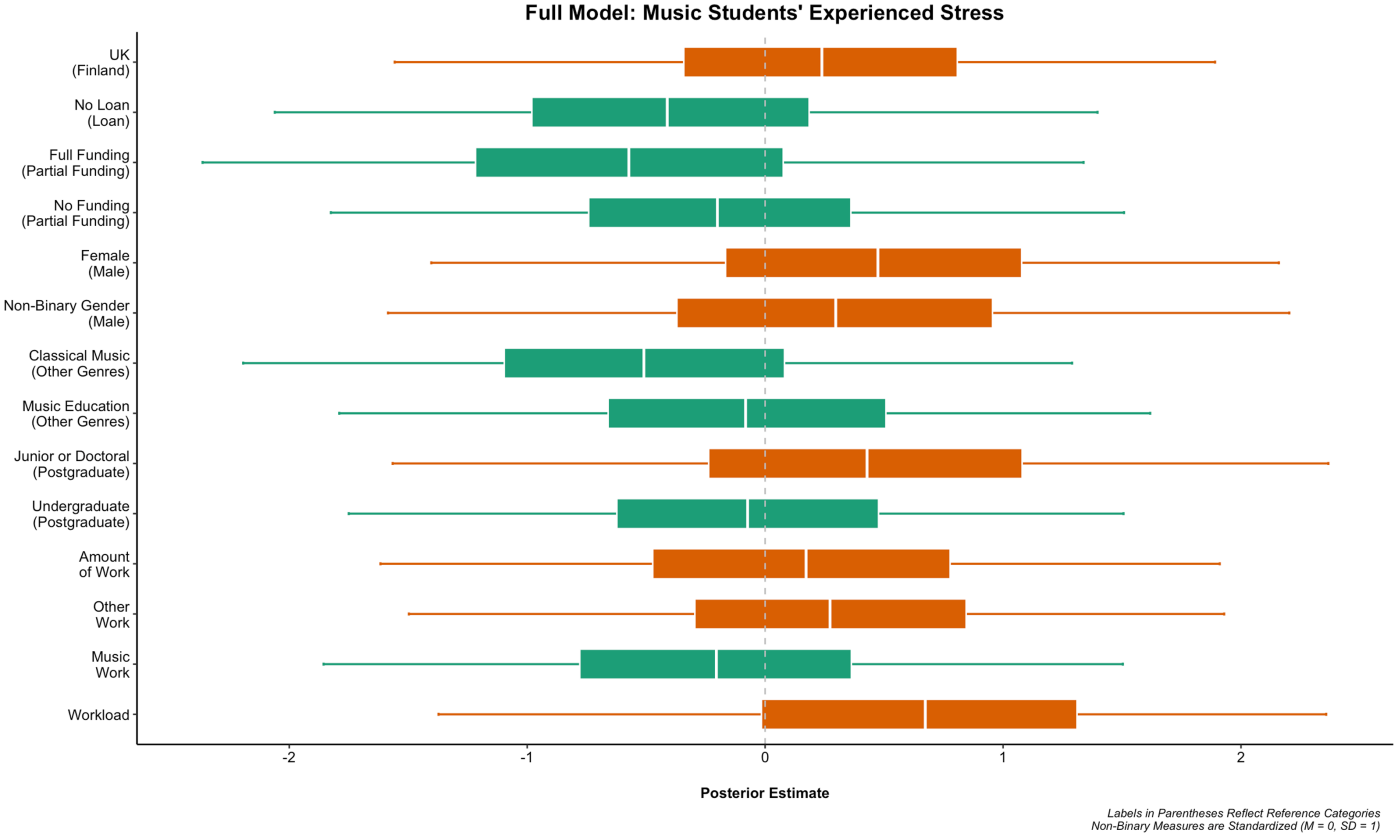
Population-level predictors of experienced stress, derived from a Bayesian mixed-effect probit regression. The boxes denote 50% posterior intervals and the lines denote 95% posterior intervals. With binary items, the left-hand side boxes indicate a smaller effect on stress than in the reference group in brackets, and the right-hand side boxes indicate a greater effect on stress than in the reference group in brackets. With the working and workload items (the four bottom items on the figure), the left-hand side boxes indicate a negligible effect on stress and the right-hand side boxes indicate the greater effect on stress the nearer the box is to the right-hand side.

*“But contact teaching is sometimes very hard, if the allowed amount of absences is strict. Unfortunately, many students have to work, both for their prestige and CV, for their artistic career, or to earn extra income, although working is not recommended whilst studying*.*”*

**Figure 8:**
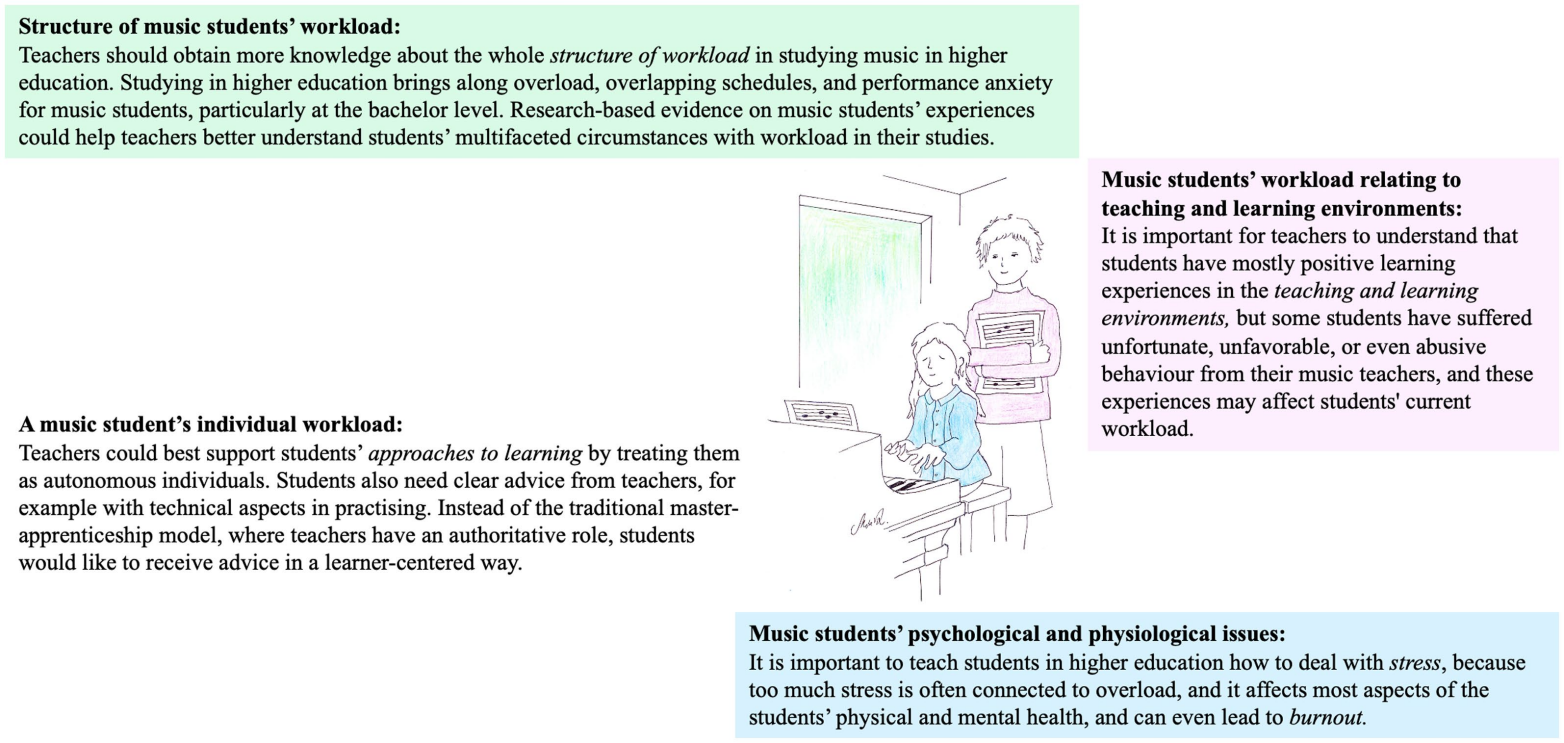
Examples of recommendations in four categories as tools for teachers to support music students in managing and coping with their workload in higher education.

In the fourth empirical study (i.e., Jääskeläinen and López-Íñiguez, 2022), based on music students’ experiences, four themes emerged in the synthesis process as recommendations for tools for teachers to support music students in managing and coping with their workload in higher education. The findings included a total of 43 recommendations. Figure 8 shows one example of these recommendations for each theme. This participant’s quotation illustrates an experience of music student’s workload in interaction with teachers:

“Even if you were not prepared well for that lesson, or even if you were extremely well-prepared, that does not matter, because when the teacher is motivated and interested in teaching you and listening to you, then you as a student are ready to work hard for learning.”

### An appraisal phase

In the appraisal phase, we critically appraised the key elements for their validity and relevance to policy and intervention recommendations. Next, the key elements for policy and intervention recommendations in each study are presented.

1. Theoretical study: Misconceptions about equity in higher music education should be revealed—for example by investigating music students’ experiences of their workload and stress as they try to cope with the demands of higher education studies—to raise and maintain a critical debate about the role of tuition fee systems, as they are connected with the economics of higher education, and about entrance examinations as reproducing social class inequalities (Jääskeläinen, 2021).
2. Systematic review: Good practices are needed (a) to increase music students’ ability to cope with their workload; (b) to provide tools for teachers to support music students to manage and cope with workload; and (c) to develop learner-centered environments in higher music education (Jääskeläinen et al., 2022b).
3. Methodological study: A practical research-based model is needed in order to process and incorporate music students’ feedback into future administrative and teaching developments in higher music education institutions, such as addressing students’ experiences in relation to curricula-related improvements (Jääskeläinen, 2022b).
4. First empirical study: An understanding of music students’ lived experiences of studying music should be utilized to improve the learning and teaching environments of institutions, and also to better support music students’ wellbeing, learning, and future careers (Jääskeläinen, 2022a).
5. Second empirical study: Studying music has its unique characteristics compared to other fields in higher education, and exploring professional music students’ particular ways of using proactive coping styles could result in valuable models for students to better cope with their studies in higher music education (Jääskeläinen et al., 2022a).
6. Third empirical study: Attention should be paid to certain aspects in higher music education in relation to workload, such as the differences between study programs and the gap between well-off students compared to low-income students who need to work, and stress, particularly with female and non-binary gender students (Jääskeläinen et al., 2020).
7. Fourth empirical study: The emphasis on music students’ workload experiences could offer a way to strengthen students’ voices so that they will be integrated into the development of teaching that trends toward more democratic practices between master and apprentice in higher music education (Jääskeläinen and López-Íñiguez, 2022).

### A synthesis phase

In the synthesis phase, we prepared a narrative account of included key elements for policy and intervention recommendations through a multiple triangulation approach (Denzin, 1970): (1) investigator triangulation in the collaboration between researchers from Finland and the United Kingdom; (2) theory triangulation by combining theories from educational psychology and music education; (3) data triangulation with two different datasets (literature and human experiences); and (4) methodological triangulation by using both quantitative method (with frequentist and Bayesian statistics) and qualitative method. In addition, triangulation helped to explain the results and findings of the studies (Noble and Heale, 2019). For example, systematic review and empirical studies complemented each other, and using multiple methods in the empirical studies led to the same concerns of differences between genders and genre groups in the music students’ experienced workload and stress.

**Figure 9:**
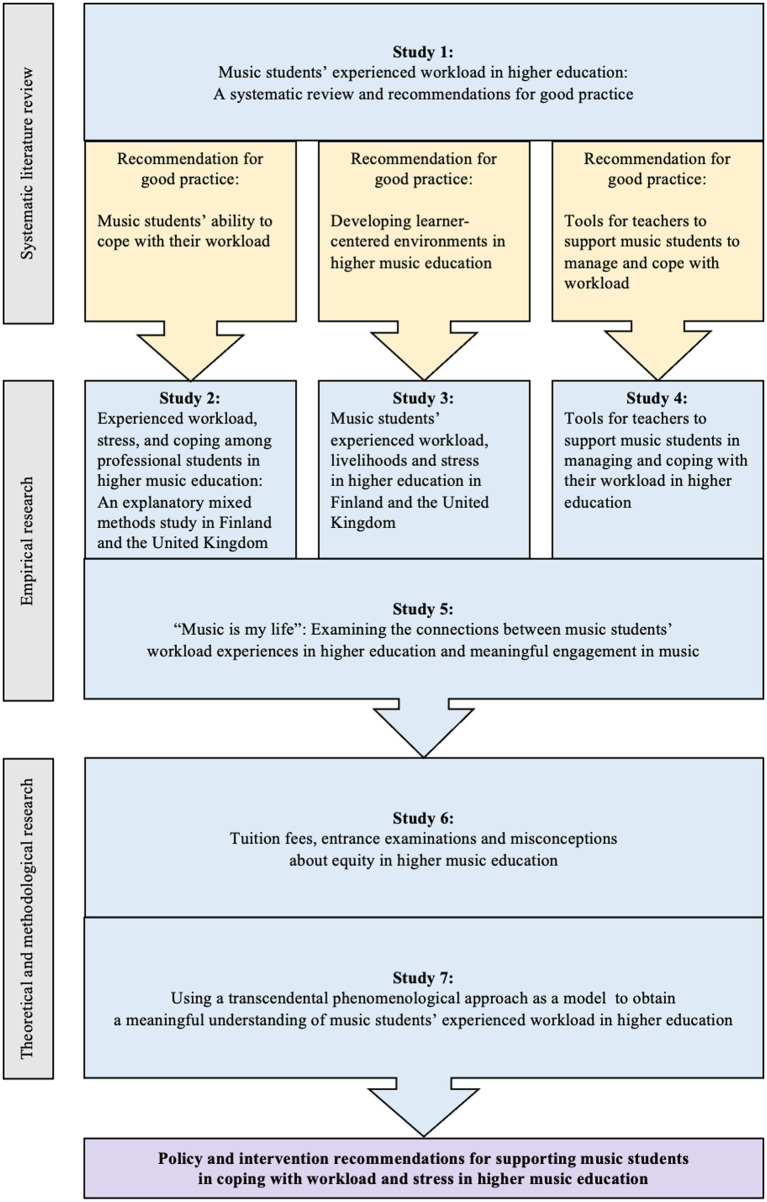
Triangulation of the seven studies in the MSW Project to create evidence-based policy and intervention recommendations.

Figure 9 illustrates how, in the synthesis phase, the recommendations for good practice, which were derived from the systematic review, further distilled the findings and results from the empirical studies in the MSW Project. It was important to triangulate the seven studies in this way to be able to construct the meta-narrative review by complementing the previous research with the new empirical results and findings regarding music students’ lived experiences. This also provided the possibility of strengthening the music students’ voices before continuing to build policy and intervention recommendations through the theoretical study and methodological study, in order to better support music students with their experiences of workload, stress, and coping in higher education. In addition, the triangulation helped to compare the multiple sources (Noble and Heale, 2019). Thus, we were able to conclude that the seven studies in the MSW Project had no conflicting results and findings.

### A recommendation phase

In the recommendation phase, we summarized the findings from the triangulated key elements, and we made policy and intervention recommendations for supporting music students in coping with workload and stress in higher music education. The recommendations represent triangulation in that each of them consists of studies including data from two sources (literature from the systematic review and human experiences from the empirical studies). These four recommendations are presented here and discussed more in detail in Discussion section:

- Recommendation 1: Support music students’ proactive coping skills (Jääskeläinen et al., 2022a,b).
- Recommendation 2: Find solutions to the unequal workload and stress experiences between low-income and well-off students, different genders, and different study programs (Jääskeläinen et al., 2020, 2022a,b).
- Recommendation 3: Ensure teachers’ continuing professional development, particularly in the learner-centered pedagogical approaches (Jääskeläinen et al., 2022b; Jääskeläinen and López-Íñiguez, 2022).
- Recommendation 4: Invest resources for providing more longitudinal, cross-cultural, and interventional research investigating music students’ discipline-specific experiences of workload and stress (Jääskeläinen et al., 2020, 2022a,b; Jääskeläinen, 2021, 2022a,b; Jääskeläinen and López-Íñiguez, 2022).

## Discussion

In the MSW Project, the aim was to view music students’ experienced workload as a very human phenomenon and an everyday issue. When the viewpoint was connected to educational arrangements and inequalities, it offered a way to lighten the load placed solely on music students’ shoulders and also to address concerns about students’ workload as a consequence of higher education practices and policies. In the present study, we adapted the six phases of the meta-narrative review according to Greenhalgh et al. (2004, 2005). As a result, the synthesis of the seven studies provided four actionable policy and intervention recommendations that will allow higher music education institutions to better support music students’ abilities to cope with their workload and stress.

### Recommendation 1: Support music students’ proactive coping skills

Students’ study skills and wellbeing could be developed through institutional practices that support students in using positive coping strategies that minimize their distress and maladaptive coping during their studies (Deasy et al., 2014). Studying music has its own unique characteristics compared to other fields in higher education (Jääskeläinen et al., 2022b). For example, the ability to cope with stress connected to stage fright is particularly important for music students (Nogaj, 2017). In our study (Jääskeläinen et al., 2022a), we showed examples of how music students use proactive coping styles: proactive coping, reflective coping, strategic planning, preventive coping, instrumental support seeking, emotional support seeking, and avoidance coping (Greenglass, 2002). Our results indicated that music students’ study workload is a significant predictor of stress; however, proactive coping and strategic planning can be used to reduce this stress. In addition, the more music students use either a proactive coping style or a reflective coping style, the less they experience study workload. It is worth noticing that in our study, female students reported experiencing statistically significantly more stress than male students; however, male students reported using the proactive coping style statistically significantly more than female students did. In addition, non-binary gender students reported using the emotional support-seeking coping style statistically significantly less than female and male students, which raises the concern that non-binary gender students may not find suitable support for themselves in the current educational structures. This confirms the fact that music students’ proactive coping skills should be better supported in order to help them manage stress and workload in their studying.

### Recommendation 2: Find solutions to the unequal workload and stress experiences between low-income and well-off students, different genders, and different study programs

To tackle the negative impact of university culture on students’ wellbeing, and to be able to acknowledge students’ diverse backgrounds and circumstances while studying (Jääskeläinen et al., 2022b), higher education institutions should utilize research on music students’ health (Williamon and Thompson, 2006; Ginsborg et al., 2009) and consider certain actions, such as shifting the typically competitive atmosphere toward a more cooperative university culture (Fernández-Herrería and Martínez-Rodríguez, 2016; Fitzpatrick, 2019) and developing the study programs by utilizing more diverse sources of knowledge (Cannella and Koro-Ljungberg, 2017). Our study (Jääskeläinen et al., 2020) of music students’ workload and stress, for which the country of study and music students’ livelihoods were combined as results predictors, suggested that a neoliberal university culture with high tuition fees that impacts students’ livelihoods alongside their studying is likely to increase music students’ experienced stress, but not directly impact on the workload associated with their main subject studies. However, participants’ lived experiences confirmed the fact that stress has a great effect on students’ experiences of their workload (Jääskeläinen et al., 2020, 2022a). These results and findings show differences in workload and stress between genders and study programs, and emphasize a particular concern over the unequal workload and stress experiences between low-income and well-off students. These differences should be further investigated with regard to the connection between higher music education systems and their curricula, in order to identify the reasons why these unequal differences exist and determine solutions for how study programs could be developed to have an appropriate and equal workload for all music students.

### Recommendation 3: Ensure teachers’ continuing professional development, particularly in the learner-centered pedagogical approaches

The emphasis on researching music students’ workload experiences offers a way to strengthen students’ voices so that they can be integrated into developmental work in teaching (Jääskeläinen et al., 2022b). This research-based evidence could be utilized to shift teacher-centered and product-oriented methods of teaching music toward learner-centeredness in teacher training that promotes “a deep understanding based on the integration of students’ prior knowledge and curricular outcomes, as well as helping students to take metacognitive control of their own learning” (López-Íñiguez et al., 2014, p. 158). In our study (Jääskeläinen and López-Íñiguez, 2022), we presented tools for teachers that would enable them to support music students in coping with their workload and stress by taking a constructive approach that would provide more spaces for learner-centeredness and agency (see, e.g., Pozo et al., 2022). Such practical tools may particularly help those teachers and students who experience difficulty in consciously accessing their cognitive and metacognitive processes when the stability and internalization of their conceptions make them strongly resistant to change (Atkinson and Claxton, 2000; Pozo et al., 2006). The research-based knowledge on students’ workload, stress, and coping as part of the teachers’ continuing professional development, when connected to the principles of conceptual change (in line with Vosniadou, 2013), can support the development of instruction toward more democratic practices between master and apprentice in higher music education (see, e.g., Gaunt and Westerlund, 2013).

### Recommendation 4: Invest resources for providing more longitudinal, cross-cultural, and interventional research investigating music students’ discipline-specific experiences of workload and stress

It is particularly important to invest resources for producing research-based evidence on the different aspects of music students’ experienced workload and stress because these experiences are different from the workloads in other fields (Jääskeläinen et al., 2022b); for instance, music students’ responsibilities include demonstrating their musical progress and managing their own coursework (Bernhard, 2007). According to Hallam and Papageorgi (2017), it is also important to nurture music students’ love and enjoyment of music alongside their studies. In line with this sentiment, in our study (Jääskeläinen, 2022a), we provided valuable insights into what engaging in music means to music students in relation to their experienced workload during their studies in higher education. Our results align with the growing need to conduct more research on students’ emotional wellbeing in higher music education (Ginsborg et al., 2009; Araújo et al., 2017), particularly attending to minority and marginal groups (Beban and Trueman, 2018) as became evident in our empirical results of non-binary gender music students who reported using less emotional support seeking coping style than female and male students (Jääskeläinen et al., 2022a) and experiencing stress but not workload in their main subject studies (Jääskeläinen et al., 2020). This may bring to light new knowledge that may better inform future administrative, teaching, and curriculum developments in higher music education (Jääskeläinen, 2021; Jääskeläinen and López-Íñiguez, 2022). In our study (Jääskeläinen, 2022b), we also presented the detailed analytical steps in transcendental phenomenology as a practical model for music institutions to gather and analyze this kind of very specific qualitative data of music students’ workload experiences during their studies. We also showed in our study (Jääskeläinen et al., 2020) how in the context of higher music education institutions, where the study programs are quite small, a Bayesian approach is a good option for analyzing quantitative data because it can produce valid results for small samples and combine both quantitative and qualitative feedback from students (Low-Choy et al., 2017). Thus, the multi-strategy approach utilized in the MSW Project may offer a valuable model of how to utilize students’ feedback in the most beneficial way to feed into developmental work in universities and educational policies. Furthermore, institutions should employ a researcher who is able to conduct longitudinal, cross-cultural, and interventional research on music students’ workload, stress, and coping, and to incorporate students’ feedback as accurate results and findings to contribute to the developmental work in the institutions.

### Theoretical implications

Because research-based findings regarding music students’ experienced workload in higher education were lacking to a great extent, the findings and results of the theoretical, methodological, and empirical studies in the MSW Project showed specific developmental areas—in line with the results of the systematic review—which were further reflected in the four recommendations above. The meta-narrative synthesis shows that the higher education context for music students has specific challenges and resources associated with music students’ coping with workload and stress when compared to those of students in other disciplines. These discipline-specific characteristics should be acknowledged in general educational theories concerning students’ workload to better inform the educational practices and policies which have an impact not only on music students’ coping, learning, wellbeing, and future careers as musicians but also on the preparedness of higher music education teachers and the development of more equitable and just education systems.

### Practical implications

When aiming to support music students’ coping with their workload and stress experiences, higher music institutions should concentrate on scrutinizing the workload in the current educational systems, for example in the curriculum and the academic culture. Student workload has been an ongoing topic in higher education for decades; however, discussing is not enough when there is growth in students’ psychological distress. Urgent actions are needed, for example by following the present study’s practical implications:

1. Listen to music students as experts of their experiences so that the support systems that help students cope with workload and stress can function properly, both in future times of crises and in everyday circumstances.
2. Recruit an institutional researcher to conduct longitudinal, cross-cultural, and interventional research on music students’ experiences of workload, stress, and coping.
3. Invest resources to disseminate research-based evidence on music students’ coping with workload and stress to inform the curricular, administrative, and teachers’ and students’ professional development.

### Limitations

The MSW Project is not without limitations. The included studies in the systematic literature review were mostly from Western countries, and the participants in the empirical studies were from only two countries, which limits the generalizability of the findings for other countries. In addition, extending the statistical representativeness of sample sizes in the empirical studies would increase the results’ generalizability. Another limitation concerns the use of self-reported experiences by music students. Biophysical data of students’ workload and stress could complement self-reported data (see, e.g., Asikainen and Katajavuori, 2021), and thus increase the validity of workload research for building educational policy recommendations.

### Future research

Because, before the MSW Project, there was practically no previous research on music students’ workload experiences in higher music education, more research is needed on this specific topic in order to guide and inform curricular decisions (Koops and Kuebel, 2021). This may help to foster an academic culture that encourages more positive learning environments as a way to meet the specific needs of music students (Papageorgi et al., 2010). Similarly, Blackwell et al. (2020) argue that research-based evidence on teaching conditions that increase students’ experiences of wellbeing may be an efficient way to improve teaching and learning environments in higher music education.

### Conclusion

In the MSW Project, we listened to music students’ experiences and showed how their valuable voices can contribute to a wide spectrum of knowledge and become a form of research-based evidence that can potentially be utilized in better supporting students and furthering both the development of academic practices and educational policies. A meta-narrative review of the seven studies in the MSW Project was conducted to map previous and new research on music students’ experienced workload. We reviewed the key elements of the findings and results from these seven studies. After triangulating the key elements, we built a synthesis to provide four actionable policy and intervention recommendations for supporting music students in coping with workload and stress in higher music education: (1) support music students’ proactive coping skills; (2) find solutions to the unequal workload and stress experiences between low-income and well-off students, different genders, and different study programs; (3) ensure teachers’ continuing professional development, particularly in the learner-centered pedagogical approaches; and (4) invest resources for providing more longitudinal, cross-cultural, and interventional research investigating music students’ discipline-specific experiences of workload and stress. The present study has several implications. Higher music education institutions should acknowledge that higher education context has specific challenges and resources associated with music students’ coping with workload and stress; thus, music students should be listened as experts of their experiences. In order to incorporate students’ feedback as accurate results and findings to the developmental work in the institutions, a researcher should be recruited and resources should be invested to produce and disseminate research-based evidence on music students’ coping with workload and stress.

## Author contributions

The author confirms being the sole contributor of this work and has approved it for publication.

## Funding

This work was funded by the Sibelius Academy Music Education Doctoral Programme and supported by the Center for Educational Research and Academic Development in the Arts (CERADA), University of the Arts Helsinki, Finland.

## Conflict of interest

The author declares that the research was conducted in the absence of any commercial or financial relationships that could be construed as a potential conflict of interest.

## Publisher’s note

All claims expressed in this article are solely those of the authors and do not necessarily represent those of their affiliated organizations, or those of the publisher, the editors and the reviewers. Any product that may be evaluated in this article, or claim that may be made by its manufacturer, is not guaranteed or endorsed by the publisher.
